# Supplementary figures and images for: The evolution of a new cell type was associated with competition for a signaling ligand
Source: PLoS Biol. 2019 Sep 18;17(9):e3000460. doi: 10.1371/journal.pbio.3000460 (PMC6768484; doi:10.1371/journal.pbio.3000460)

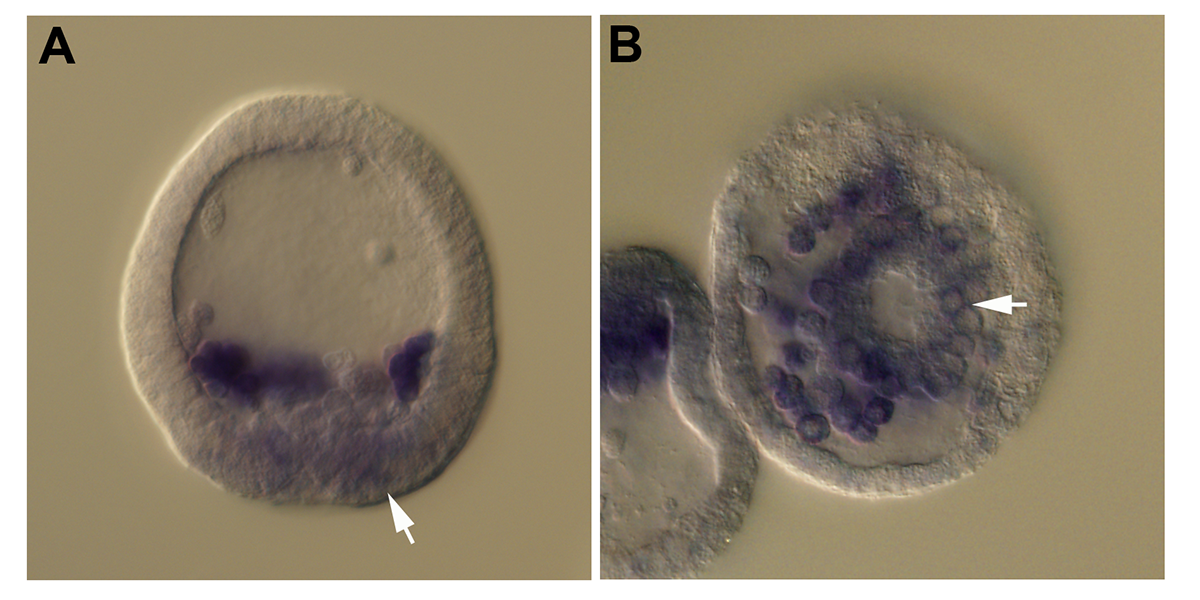

Supplement: S1 Fig — Whole-mount in situ hybridization analysis of Lv-vegfr-10-Ig expression in control, early gastrula-stage embryos. (A) Lateral view. (B) Vegetal view. Expression in the wall of the archenteron (presumptive nonskeletogenic mesoderm) is indicated by arrows. Lv-vegfr-10-Ig, L. variegatus vascular endothelial growth factor receptor-10-Ig. (TIF) [file pbio.3000460.s001.tif]

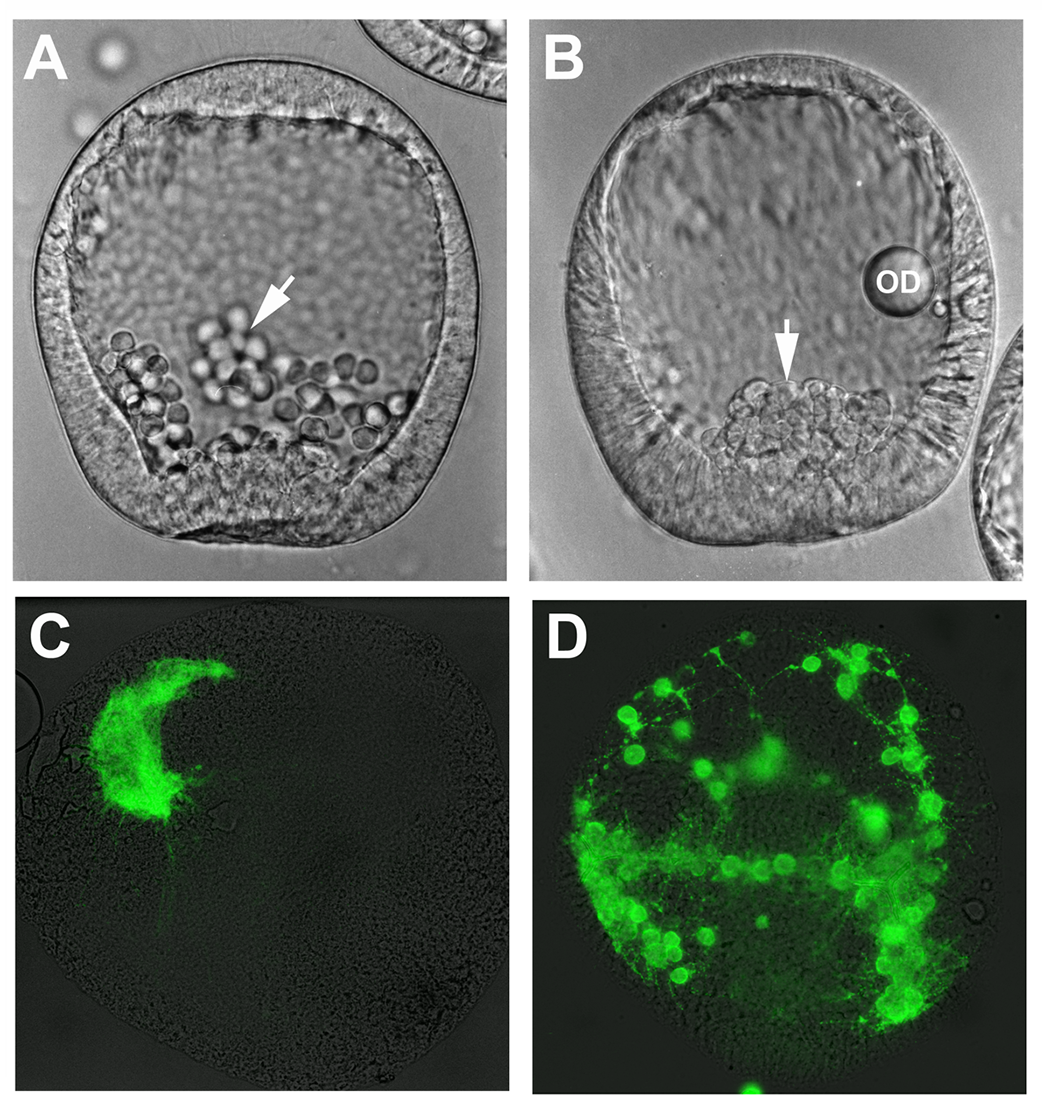

Supplement: S2 Fig — (A) Control early gastrula-stage embryo. PMCs (arrow) have dispersed from the site of ingression and are migrating along the blastocoel wall. (B) Sibling embryo, 4 hours after microinjection of WGA into the blastocoel. The PMCs (arrow) remain in a single mass at the site of ingression. The embryo is marked with an OD. (C) WGA-injected embryo 12 hours after injection, immunostained with 6a9 antibody. WGA has caused the coalescence of the PMCs into a single large mass from which numerous filopodia extend. No other 6a9(+) cells are present at the tip of the archenteron or in the blastocoel, indicating that BC transfating has not occurred. Analysis of WGA-injected, 6a9-stained embryos at multiple stages during gastrulation confirms that no BC cells transfate when PMC migration and patterning is disrupted. (D) PMC(−) embryo injected with WGA immediately after PMC removal and immunostained with antibody 6a9 after 12 hours. Numerous 6a9(+), transfated BCs are apparent, demonstrating that BCs are capable of transfating in the presence of WGA. BC, blastocoelar cell; OD, oil droplet; PMC, primary mesenchyme cell; WGA, wheat germ agglutinin. (TIF) [file pbio.3000460.s002.tif]
