# Supplementary material for: The evolution of a new cell type was associated with competition for a signaling ligand
Source: PLoS Biol. 2019 Sep 18;17(9):e3000460. doi: 10.1371/journal.pbio.3000460 (PMC6768484; doi:10.1371/journal.pbio.3000460)
Supplement: S1 Data — Raw cell counts for all experiments presented in this paper. Each value represents the number of 6a9-positive cells in a single embryo. The data are shown graphically in the specific figure panels indicated. (DOCX) [file pbio.3000460.s003.docx]

**Supplemental File 1- Counts of 6a9-Positive Cells**

Shown below are the raw cell counts for all experiments presented in this paper. Each value represents the number of 6a9-positive cells in a single embryo. The data are shown graphically in the specific figure panels indicated.

**Fig. 2H**

Trial 1

Controls (no axitinib)- 59, 72, 72, 65, 66, 64, 58, 64, 78, 71

Axitinib-treated embryos- 4, 3, 1, 0, 3, 5, 8, 1, 10, 3, 0

Trial 2

Controls (no axitinib)- 62, 54, 94, 42, 64, 48, 55, 59, 61

Axitinib-treated embryos- 1, 8, 0, 4, 6, 3

**Fig. 3B**

Controls (no axitinib)- 59, 72, 72, 65, 66, 64, 58, 64, 78, 71, 62, 54, 94, 42, 64, 48, 55, 67, 58, 56, 64, 50, 39, 72, 83, 80, 51, 51, 83, 62, 66, 74, 60, 70, 52, 51, 54, 79, 96, 76

Axitinib added 0-1 hpd- 4, 3, 1, 0, 3, 5, 8, 1, 10, 3, 0, 2

Axitinib added 2-3 hpd- 1, 11, 5, 0, 4, 10, 7, 0, 0, 2, 4, 5, 1, 8, 0, 4, 6, 3

Axitinib added 3-4 hpd- 6, 11, 5, 24, 2, 7, 7, 17, 26, 11, 4, 8, 3, 3, 6, 0, 7, 10, 8, 4, 6, 5

Axitinib added 4-5 hpd- 10, 36, 26, 20, 5, 20, 24, 22, 24, 12, 10, 18, 20, 28, 20, 20, 7

Axitinib added 5-6 hpf- 46, 30, 52, 35, 40, 44, 50, 43, 30, 59, 48, 30, 46, 62, 46, 36

**Fig. 5F**

Trial 1

Controls- 88, 52, 34, 65, 108, 48, 82, 48, 58, 78

VEGFR Morphants- 32, 31, 5, 34, 29, 33, 29, 8, 16, 17, 27, 28, 17, 13, 33, 19, 17

Trial 2

Controls- 58, 56, 69, 64, 48, 72, 52, 40, 34, 64, 50, 68

VEGFR Morphants- 10, 8, 6, 5, 6, 12, 7, 8, 8, 6, 6

Control

Controls- 52, 30, 57, 36, 49, 47, 22, 70, 60, 80, 60, 56, 64

IgTM Morphants- 28, 44, 50, 68, 66, 32, 76, 81, 23, 29, 49, 67, 42, 43, 85, 42, 81, 81, 73

**Fig. 6D**

Trial 1

Controls- 40, 60, 48, 65, 65, 51, 68, 48, 62, 56, 42, 54, 44, 36, 56, 40, 75, 26, 67, 65, 73, 46

VEGF Morphants- 10, 14, 6, 35, 24, 15, 11, 4, 17, 18, 3, 13, 20, 4, 8, 1, 20, 1, 16, 42, 6, 6, 4, 0, 40, 6, 10

Trial 2

Controls- 52, 26, 27, 30, 45, 50, 30, 40, 60

VEGF Morphants- 14, 5, 4, 3, 34, 0, 12, 9, 8, 8

**Fig. 7F**

See Supplemental File 2

**Fig. 8D**

Controls- 39, 43, 42, 43, 34, 45, 65, 40, 60, 42, 56, 50, 54, 51, 37, 53, 54, 45, 50, 33, 44, 59, 55, 51, 57, 52, 52, 50, 53, 42, 38, 40, 38, 37, 50, 42, 40, 44, 43, 45, 54, 70, 34

0.05 mg/ml VEGF mRNA- 56, 52, 47, 49, 75, 47, 46, 42

0.1 mg/ml VEGF mRNA- 54, 50, 44, 48, 51, 60, 56, 46, 54, 54

0.2 mg/ml VEGF mRNA- 44, 54, 41, 54, 48, 66, 61, 66, 54, 60, 58, 42, 46, 52, 68

0.4 mg/ml VEGF mRNA- 45, 39, 83, 92­, 83, 61, 68, 48, 61, 68, 61, 37, 42, 43, 45, 74, 50, 39

0.8 mg/ml VEGF mRNA- 84, 68, 102, 93, 46, 50, 54, 56, 90, 76, 58

1.6 mg/ml VEGF mRNA- 60, 60, 42, 84, 60, 86, 106, 54, 80, 70, 110, 72, 56

3.2 mg/ml VEGF mRNA- 72, 92, 58, 58, 64, 86, 66, 84, 80, 84, 65, 54, 52, 80, 66, 68, 76, 70, 78, 72, 96, 89
