# Supplementary material for: The evolution of a new cell type was associated with competition for a signaling ligand
Source: PLoS Biol. 2019 Sep 18;17(9):e3000460. doi: 10.1371/journal.pbio.3000460 (PMC6768484; doi:10.1371/journal.pbio.3000460)
Supplement: Attachment — Submitted filename: Response to Reviewers (PLOS Biology MS).docx [file pbio.3000460.s005.docx]

Response to Reviewers

We thank the four reviewers for their careful evaluations of the manuscript and for their valuable recommendations. We have made several significant changes in response to their comments and feel that the revised manuscript is significantly improved. Below are point-by-point responses to the reviewers’ concerns:

**Reviewer 1**

1) “*It is not clear how VEGF3 ligand is received by the non-skeletogenic cells as VEGFR-10-IG is expressed only in the skeletogenic cells in normal embryos…I think more effort should be made to try and identify the missing receptor. The authors should try and see if they can detect VEGFR-10-IG in the non-skeletogenic cells either by overstaining WMISH or by antibody for the protein, if available. I also think that WMISH of VEGFR7 would be relevant…Another possibility is that at high concentrations VEGF3 could be acting through another RTK receptor. All these possibilities should be mentioned and more effort should be put in studying the spatial expression of VEGFR-10-IG and VEFGR7 in the non-skeletogenic mesoderm and endodermal cells (WMISH).”* 

In response to the Reviewer’s comments we decided to go back and look more closely at the normal expression pattern of Lv-vegfr-10-Ig. Surprisingly, we’ve observed low levels of expression in presumptive non-skeletogenic mesoderm cells in the wall of the archenteron at the early gastrula stage. This was unexpected based on the previously published data in *P. lividus* (Duloquin et al., 2007) but may have been missed earlier because the expression is much lower than in PMCs and is also transient (it is apparent only at the early gastrula stage). We present these WMISH data in a new figure (SI Fig. 1) and in the Results, L135-141. Although unfortunately we do not have a good antibody against the VEGFR-10-Ig protein, we feel these new mRNA expression data are extremely valuable. They support the view that non-skeletogenic mesoderm cells are receptive to VEGF3 because they normally express low levels of the receptor. We thank the Reviewer for suggesting that we perform these studies.

We have not carried out WMISH studies on VEGFR7 because the expression level (< 40 transcripts/embryo at the mesenchyme blastula stage) (Tu et al., 2014) is below the detection limit of WMISH, even if the mRNA is entirely restricted to presumptive BCs.

2) *“I do not understand why throughout the paper the authors refer only to the blastocoelar cells when the cells that ectopically express Alx1, VEGFR-10-IG and become skeletogenic include also endodermal cells and possibly other non-skeletogenic mesodermal fates.”*

The reviewer is correct that endoderm cells also possess skeletogenic potential, but they only exhibit this potential under specific experimental conditions. Here we focus on BCs because our experimental paradigm throughout the paper is the removal of PMCs (except for the VEGF overexpression experiment). Under these conditions, only BCs transfate, not other cells. Transfating of endodermal cells occurs only after removal of the tip of the archenteron (which includes the BCs) in addition to the PMCs. On a side note, we showed previously that reprogramming of endoderm cells after PMC + archenteron removal is associated with the re-specification of BCs, so it’s possible that endoderm cells transfate via an intermediate, BC-like state (Sharma and Ettensohn, 2011).

3) “*I …believe that the statement that VEGF-signaling controls Alx1 expression at the late sea urchin embryo is not correct and should be removed from the paper.”*

The reviewer is correct that at very late stages of embryogenesis, *alx1* is selectively expressed in the scheitel region in response to a signal distinct from VEGF3. Our reference here was based on *alx1* expression at the late gastrula stage, when the gene is selectively expressed in PMCs on the ventral side of the embryo, where VEGF is also expressed (Sun and Ettensohn, 2014, Fig. 2, late gastrula, Class II genes). Morgulis et al. (2019) also document a modest reduction in *alx1* expression in axitinib-treated embryos (Fig. S6, P, 30 hr). We agree with the reviewer, however, that this constitutes rather weak evidence and therefore have qualified our statements on this point (L352-354, 738-740).

4) *“The common transcription factors expressed in both skeletogenic mesoderm (SM) and non-skeletogenic mesodermal (NSM) cells could be a key to the potential of the NSM to become SMs, and I think that more details should be provided about it in the manuscript.”*

We agree strongly with the Reviewer that this is a distinct possibility. In the new version of the manuscript, we have significantly revised and expanded the relevant section of the Discussion (L325-343). We now specifically mention Ets1 and Erg, but not Tel or Hex, only because we feel the published WMISH data regarding the latter two genes aren’t as clear.

5) *“I think it would be interesting to discuss the findings of this paper in the light of two recent publications that studied the role of VEGF in sea urchin skeletogenesis (Morgulis et al 2019) and in brittle star skeletogenesis, where FGF signaling seem to have taken a lead role instead of VEGF signaling… Specifically, in the last paragraph of the paper that discusses the role of VEGF in skeletogenesis in echinoderms.”* 

We thank the reviewer for highlighting the Morgulis et al 2019 paper, which was published after we submitted our original manuscript. We now refer to this paper in the Introduction (L50-53). We would prefer not discuss the brittle star work in detail, however, until it has undergone peer-review.

6) *“The experiment that shows that VEGFR-10-IG turns on when protein translation is blocked is very interesting (Fig. 4). I wonder if they did the same experiment with Alx1 – does the initial transcriptional response mediated by proteins that are already present in these cells?”* 

No, we have not tried the same experiment with *alx1*. We believe that our experiment with VEGFR-10-Ig, however, shows that the rapid transcriptional response of this gene is mediated by proteins already present in the cell.

7) *“The experiment where the authors return PMCs to the skeletogenic-depleted embryos is beautiful (Fig. 7), but I don't understand how come the fluorescence dye from the donor PMC doesn't diffuse to the transfated cells in the recipient embryo.”*

Transfated cells do fuse with PMCs and soluble, cytoplasmic markers (like GFP or fluorescent dextran) spread rapidly throughout the syncytium. In this experiment, however, PMCs were labeled by bathing embryos in RITC, a reactive dye that covalently attaches the fluorochrome to exposed lysine residues on PMC surface proteins. These rhodamine-tagged cell surface proteins do not diffuse readily from cell body to cell body, even after fusion. We have added a statement clarifying this important point (L211-215).

8) *“The experiment where the authors injected WGA to the embryos to show that VEGFR phenotypes are not due to defects in migration is only described in words. Representative images of this experiment would make it more visual and understandable, maybe as a sup figure.”*

We have added a new figure (SI Fig. 2) showing these results, and thank the Reviewer for the suggestion.

9) “*The first paragraph of the discussion starts with a very general statement and the main point is only presented in the sentence before the last (the sequestering mechanisms). I think this paragraph should start with a stronger, more focused statement about sequestering mechanisms, and then define the gap in knowledge that this paper addressed. I think it would lead better to the rest of the discussion.”* 

We agree with the Reviewer and have replaced (and hopefully strengthened) the first sentence of the Discussion (L256-257).

10) *“The methods section is very brief and a description of the emetine protocol is missing.”*

It’s true that the Methods section is rather short, but this is primarily because most of the experimental approaches have been described in detail in previous publications. We have added a detailed description of the emetine protocol, and thank the reviewer for detecting this omission (L389-394). We have also added a detailed description of the WGA injection experiments (L396-402, the results of which are shown in new SI Fig. 2.

11) *“In the second paragraph of the introduction in the third line the word "also" appears twice.”*

This has been corrected.

12) *“It would be helpful to add line numbers to the text.”*

Line numbers have been added.

**Reviewer 2**

1) *“I suggest the authors to describe the alternative hypothesis with which they compare with the ligand competition model… Perhaps the alternative hypothesis is that PMCs send some inhibitory signal to BCs. The two hypotheses may not be completely exclusive, but without that information, it was not easy for me to understand why the authors need to show VEGF signaling is required in BC transfating (I felt that we already know that!).”*

We agree with the reviewer that the signal from PMCs to BCs can be thought of as an inhibitory signal, in the sense that BC transfating is blocked when PMCs are present. Therefore, in the Introduction we describe the PMC signal as a “suppressive” signal (L57). In this paper, we try to provide a molecular mechanism for the ability of PMCs to suppress the deployment of the skeletogenic GRN by BCs. We show (and this was NOT previously known) that BCs require VEGF to initiate this program (i.e., to activate *alx1* and *tbr*). We provide evidence that the mechanism by which PMCs suppress BC transfating during normal development is by out-competing BCs for VEGF during normal development. In other words, the sequestration of VEGF is the mechanism that underlies the PMC inhibitory signal.

2) *“It would be critical to show whether the expression of Alx1 and Tbr in 2 hpd is affected by VEGF signaling inhibition.”*

We agree with the Reviewer that it is important to know the time course of the axitinib effect. Although we show 4 and 5 hpd time points in Figure 2, we’ve also analyzed embryos at 3 hpd and see a very clear reduction in both *alx1* and *tbr* expression at this time. We’ve added a statement to this effect to the text (L103-104) and can include supplementary images if the Reviewer feels these are needed. By either conventional or fluorescence-based WMISH approaches, the initial expression of *alx1* and *tbr* detected at 2 hpd is weak and not apparent in all embryos, which, which makes it difficult to reliably score drug effects at this stage.

3) *“I suggest the author to design experiments in which high level expression of VEGF only in epidermal cells.”*

The reviewer is correct in stating that our VEGF overexpression experiment, which involved microinjection of VEGF mRNA into fertilized eggs, results in production of VEGF by most or all cells of the embryo, probably including BCs. We acknowledge that we cannot exclude the possibility that our experiment produces an autocrine effect of VEGF3 on BCs. Even if this occurs, however, we do not feel that it invalidates an important conclusion of these experiments; namely, that activation of the skeletogenic GRN (*alx1* and *tbr*) is triggered when VEGF ligand becomes available to the BCs. The proposed experiment is logical but technically challenging because good tools are not available in sea urchins for efficiently driving gene expression in specific cell types at late developmental stages. To produce sufficiently high levels of expression specifically by epidermal precursors, we could try microinjecting mRNA into multiple blastomeres of the 16-cell stage embryo (when the precursors can first be identified), but this is technically very difficult. The use of cell type-specific promoters to drive VEGF expression at sufficient levels is complicated by the highly mosaic expression of transgenes in sea urchins. Based on the reviewer’s comments, we have added a statement to the Discussion stating explicitly that the effect of VEGF3 mRNA might be due to autocrine signaling (L277-280) and hope this is satisfactory.

4*) “Figure 9 is not accurate for the expression of VEGFR in BC cells. VEGFR is expressed at a very low level, if any, in BC cells. Isn’t it?”*

In the revised manuscript, we present WMISH data showing that *vegfr-I0-Ig* is indeed expressed transiently and at low levels in presumptive non-skeletogenic mesoderm (SI Fig. 1). Please also see our response to Reviewer 1, Point 1.

5) *“Regarding the issue on the novel cell type evolution, I suggest the authors to clarify whether the absence of specific expression of VEGFR in BC cells are the ancestral state of echinoderms before the acquisition of PMCs.”*

In all echinoderms that have been examined (including embryonic stages and adult forms from several different taxa), high levels of *vegfr-10-Ig* expression are observed only in the skeletogenic (*alx1*-expressing) cells. Therefore, it seems very likely that elevated *vegfr-Ig-10* expression was specifically associated with the invention of skeletogenic cells very early in echinoderm evolution, long before the evolutionary appearance of PMCs in euechinoids (see also Erkenbrack and Thompson, 2019). We don’t know what the precursor cell type was like that gave rise to the first echinoderm skeletogenic cell, or the evolutionary relationship between that ancient, mesenchymal cell type and modern BCs. Maybe the best proxy we have for the ancestral BC is the late ingressing, non-skeletogenic mesenchyme of asteroids. These cells express some genes that are associated with a BC fate in euechinoids (Ets1 and Erg) but do not express *alx1* or high levels of *vegfr-10-Ig* (McCauley et al., 2010; Morino et al., 2012). This might reflect the ancestral state of BCs, although obviously extrapolating from modern species, especially based on extremely limited comparative data, is dangerous. In response to the Reviewer’s question we have tried to clarify these points in the Discussion (L344-350) and hope that we have not misinterpreted the question.

**Reviewer 3**

1) *“The only technical concern I had was the lack of any real evidence to explain how the blastocoelar cells perceive VEGF3 (or the absence of PMCs if the signal is more indirect). The authors speculate that blastocoelar cells might produce a small amount of receptor that is simply too low to detect by in situs. Given that PMCs can be removed from live embryos, is it not possible to isolate some blastocoelar cells and test this directly by qPCR?”*

In the revised manuscript, we present WMISH data showing that *vegfr-I0-Ig* is indeed expressed transiently and at low levels in presumptive non-skeletogenic mesoderm (SI Fig. 1). Please also see our response to Reviewer 1, Point 1. We did attempt to surgically isolate the tip of the archenteron (which includes the presumptive BC) at the early gastrula stage for RT-PCR analysis, but found that one or two PMCs were almost always inadvertently co-isolated along with the archenteron tissue.

2) *“A larger question that needs to be addressed more directly in the Discussion is why blastocoelar cells are still capable of responding to VEGF3 when it has apparently been hundreds of millions of years since they were the source for skeletogenic cells. … A possible explanation that the authors may have already considered is that the blastocoelar cells are actually the source population for the cells that make the adult skeleton and that the ability to differentiate into skeletogenic cells is part of normal development that simply takes place much later. Is there any evidence one way or the other to support this idea?”*

We agree strongly with the Reviewer that this is a real possibility, but we don’t yet have the necessary cell lineage data. We think it’s very possible that BCs give rise to the new skeletal structures that arise in the larva after feeding begins (i.e., the dorsal arch and posterodorsal rods). They might also contribute to the adult skeleton, although others have speculated that the coelomic pouch mesoderm forms the adult skeleton. The only relevant work we know of is the 2007 study by Yajima, which showed that when labeled secondary mesenchyme cells (a mixture of pigment cells and BCs) were transplanted into unlabeled embryos, in ~20% of cases, skeletal structures became labeled and this only occurred after feeding began. Other genetic marking experiments showed that PMCs do NOT contribute to the adult skeleton, which is presumably produced by other cells. In the revised manuscript, we discuss the Yajima paper and the possibility that BCs might normally contribute to the late larval or adult skeleton (L320-324).

3) Minor points:

*“Use of the abbreviation hpd was confusing. At first I thought this was a typo. Since it is only used a few times, it would be clearer to spell it out.”*

We now spell this out in the main text but retain the abbreviation in the figures to avoid overcrowding.

*“A likely typo: second page of Results, line 4, should alx be alx1?”*

We thank the reviewer for detecting this error, which has been corrected.

**Reviewer 4** 

1) *“From what is written in the manuscript it is not entirely clear why PMC are more capable of sequestrating VEGF3 than BC. Is it because PMC start expressing VEGFR earlier than BC can, and thus a critical part of the model is that PMC are earlier than BC and thus prevent the BC to receive a strong enough VEGF3 signal to activate the positive feedback between VEGF3 and VEGFR?”* 

Yes, this is indeed what we propose. We believe that the early and very high level of VEGFR expression by PMCs, driven by the maternal specification system, gives them a competitive advantage over BCs and results in the sequestration of the ligand. We now state this more explicitly in the Discussion (L362-367).

2) *“The evolutionary scenario, though very interesting and clear to follow, could be somewhat expanded. It seems that since BC can replace PMC in PMC(-) embryos, that these two cell types could be sister cell types. Is this supported by the phylogenetic distribution of larval/embryonic structures and phylogeny? Is the primary distinguishing characteristic of PMC that they are separate earlier from the blastoderm and then dominate the role of larval skeletogenic systems and the BC, though sister to PMC, evolved a new phenotype, that of a phagocyte? Or are the BC in other echinoderms both skeletogenic and phagocytotic? At least in asteroid larvae the mesenchymal cells are phagocytotic [this is the animal where macrophage like cells where discovered!] I think this paper will have a broader and deeper intellectual impact if the scenario in Figure 10 is a little bit expanded…”*

This question is somewhat related to Question #5 of Reviewer 3 (see above). There has been much less attention paid to BCs than to skeletogenic cells, across echinoderms and even within euechinoids. For example, we lack any comparative data regarding the expression patterns of the two key regulatory genes that are thought to control the BC/immunocyte fate in euechinoids (*scl* and *gatac*). Because the BCs of other echinoderms have not been clearly identified, their phagocytic properties are also ill-defined. The evolution of phagocytic function is also complicated by the fact that euechinoid pigment cells, another derivative of the mesoderm but with a different mechanism of specification, are also phagocytic. We do think it’s possible, as the Reviewer suggests, that the late-ingressing, non-skeletogenic mesenchyme of asteroids might be very informative here, but there has been little work done to tease apart possible subpopulations of these cells or to analyze their specification. Basically (and unfortunately), we don’t feel we can adequately address the interesting and important questions this Reviewer raises regarding the evolutionary relationship between PMCs and BCs until more is known about BCs across the phylum.
